# Prevalence of a marker of active *helicobacter pylori* infection among patients with type 2 diabetes mellitus in Lagos, Nigeria

**DOI:** 10.1186/1756-0500-5-284

**Published:** 2012-06-11

**Authors:** Aderemi Oluyemi, Ebere Anomneze, Stella Smith, Olufemi Fasanmade

**Affiliations:** 1 General Hospital, Ikorodu, Lagos State, Nigeria; 2 Health Gates Consultancy and Medical Services, Ojuelegba, Lagos State, Nigeria; 3 Division of Molecular Biology, Nigerian Institute of Medical Research, Lagos State, Nigeria; 4 Department of Medicine, Lagos University Teaching Hospital, Idi-Araba, Lagos State, Nigeria

**Keywords:** *Helicobacter pylori* infection, Stool antigen test, Diabetes mellitus, Nigeria

## Abstract

**Background:**

There appears to exist a potentially important interplay between diabetes mellitus (DM) and *Helicobacter pylori (H. pylori)* infection. Findings from previous studies have been conflicting. Only a few studies have examined the topic in a sub-Saharan African population. This study sought to determine the prevalence of *H. pylori* infection among Type 2 diabetes mellitus (T2DM) patients in Lagos, Nigeria.

**Findings:**

*H. pylori* infection was detected in 18% of T2DM patients and 13% of controls but there was no statistical significance in this difference (p = 0.52). The prevalence of *H. pylori* was neither associated with the known duration of T2DM nor was it associated with age, gender, body mass index (BMI), smoking status. T2DM was not shown to be a risk factor independently associated with risk for *H. pylori* infection (OR = 0.87, 95% CI = 0.58-1.31, p = 0.57).

**Conclusions:**

The lack of a statistical significant difference between the *H. pylori* infection rates in T2DM patients and controls suggests that the infection is not increased in T2DM. Larger studies need to be conducted to confirm the study findings.

## Background

Since the discovery of the bacterium by Warren and Marshal in 1984 [1], *Helicobacter pylori* has been shown to have a world-wide distribution. It has been estimated that up to half of the world’s population harbor the infection in their stomachs [2]. The developing world has a higher prevalence rate of infection than the developed world and it has associated with both gastrointestinal and extra-intestinal ailments [3].

An increased prevalence of *H. pylori* infection among diabetes mellitus patients was first suggested by a report from Hungary [4]. It was further supported by other reports [5,6]. The latter study [6], documented a *H. pylori* prevalence rate of 74.4% in Type 2 diabetes mellitus patients as against 50% in non-diabetic controls. Another study in Italy corroborated this finding [7]. However, other more recently published data have concluded otherwise. A large Australian study [8] showed that there was no significant difference in prevalence of *H. pylori* infection in DM versus(vr) non-DM patients. The higher prevalence of infection among normal children as opposed to those with type 1 DM [9] further strengthens this opposing argument especially as childhood is thought to be the most common period of acquisition of *H. pylori* infection.

An earlier report by Ugwu et al. [10] from Nigeria examined the possibility of such a relationship in our local environment. The study reported no significant difference in the prevalence of *H. pylori* infection between diabetics and non-diabetics (35% vr 28%, p > 0.05). The study was unique as there exists little data examining this topic in sub-Saharan populations.

The mode of diagnosis that was deployed in this study, the stool antigen testing, identifies active infection unlike the serological based modalities of diagnosis which cannot distinguish between current and previous infections [11]. This study sought to determine the prevalence of *H. pylori* infection among T2DM patients in Lagos, South-West region of Nigeria.

## Methods

The study is a hospital-based, cross-sectional survey and was conducted at the Lagos University Teaching Hospital (LUTH), which is situated in Idi-Araba, Lagos State, Nigeria. It is the largest tertiary health facility in the commercial nerve centre of Nigeria and it’s catchment area of patients is state-wide with extension to adjoining states in the South West region. After obtaining informed consent, 100 consecutive, consenting T2DM patients and 100 controls were recruited from the adult out-patient wing of LUTH. Age and gender-matched controls were selected from non-diabetic volunteers among LUTH staff, students and patients’ relations. The controls were randomly requested to participate and only consenting ones were recruited for the study. After agreeing to participate and signing informed consent forms, all respondents had standard pretested written questionnaires administered to them by medical doctors. They were then required to submit fresh stool samples which were tested for evidence of active *H. pylori* infection by stool antigen positivity with the immunoassay-based Rapid Strip HpSA^TM^ according to the manufacturer’s instructions (from Meridian Bioscience Europe)(sensitivity 96.1%, specificity 90.6%) [12].

Each of the controls had blood drawn for fasting blood glucose and those that met the minimum World Health Organization requirement for diabetes mellitus diagnosis were excluded from being controls in this study. This minimum criterion is a fasting plasma glucose of ≥ 7.1 mmol/l [13]. As diarrheic stools are not appropriate for testing with the above mentioned kit [12], this was taken as an exclusion criterion for both cases and controls. Also excluded were those who had had prior antibiotic and proton pump therapy for *H. pylori* infection.

Ethical approval was obtained from the Research and Ethics Committee of LUTH prior to the commencement of the study.Data obtained was analyzed using the statistical software Epi-info version 6. Results are expressed as means ± standard deviation and frequencies. Statistical analysis was done using the students’ t-test for continuous variables and the chi-squared(x2) for categorical data. Multivariate analyses were conducted in view of possible confounding factors.

## Findings

Both study cases and controls had similar socio-demographic and anthropometric profiles and Table 1 summarizes the parameters examined.

**Table 1:** Socio-Demographic and arthropometric data of participants with type 2 diabetes mellitus and controls in LUTH, Lagos

**Characteristics**	**Cases (n = 100)**	**Control(n = 100)**	**p value**
Age (years)	Mean 56.4 ± 10.40	Mean 54.9 ± 10.30	0.15
Sex: Male (%)	43	45	0.89
Female (%)	57	55	
Duration of Diabetes Mellitus (year)	Mean 17.41 ± 8.33	Not applicable	Not applicable
Smoking Status: No (%)	82	80	0.45
Yes (%)	18	20	
Level of Education:- None (%)	11	9	0.82
Primary (%)	13	22	
Secondary (%) Post-	23	27	
Secondary (%)	53	42	

### Prevalence of *Helicobacter pylori* infection in cases and Non-diabetic controls

*H. pylori* infection as determined by stool antigen positivity was present in 18%(n = 100) of type 2 diabetic patients and 13%(n = 100) of the controls(Table 2). There was no significant difference in overall prevalence of *H. pylori* in cases and controls (x^2^ = 0.41, p = 0.52). Of the 13 infected control subjects, the prevalence was higher in men (7/45) than in women(6/55) (15.6% vr 10.9%, x^2^ = 0.09, p = 0.75). *H. pylori*-positive control subjects were not significantly younger than *H. pylori*-negative subjects (53.6 ±12.30 vr 55.1 ±10.04 years, p = 0.84); The overall prevalence of *H. pylori* infection was 18% (n = 100) in patients with T2DM. The prevalence of *H. pylori* infection was 16.4% (7/43) in men and 21.1%(12/57) in women with T2DM (x^2^ =0.06, p =0.81). The *H. pylori*-positive type 2 DM patients were noted to be were slightly older than *H. pylori*-negative counterparts in this study but the difference was also not statistically significant (55.1 ± 9.95 vr 56.7 ± 10.53 years, p = 0.91).

**Table 2:** **Comparison of*H. pylori*status among study participants with type 2 diabetes mellitus and controls in LUTH, Lagos**

	**Cases(n = 100)**	**Controls(n = 100)**	**Total(=200)**
***H. pylori*POSITIVE**	18	13	31
***H. pylori*NEGATIVE**	82	87	169

The prevalence of *H. pylori* infection was not associated with the known duration of T2DM. The prevalence of the infection was 26.9% (7/26) in patients with T2DM diagnosed ≤ 2 years prior, 20.8% (5/24) in those diagnosed 2–5 years prior, and 14% (7/50) in those diagnosed ≥5 year duration(x^2^ = 1.25, p = 0.52). There were 24 obese T2DM in this study as against 17 in the control group. *H. pylori* was positive in 12.5% of obese diabetics (3/24) and 19.7% (15/76) of diabetics with normal BMI (x^2^ = 0.14, p = 0.74). There was no association between *H. pylori* infection and smoking history; the prevalence of the infection was 32.5% (66/203) in those with a previous or current smoking history and 31.1% (68/219) in non-smokers (x^2^ = 0.02, p = 0.89).

In patients with diabetes and control subjects, logistic regression analysis (including age, gender, diabetes status, BMI, level of education and smoking history) revealed that not any one of the above mentioned factors was independently associated with *H. pylori* infection.

## Discussion

### Prevalence of *H. pylori* infection in type 2 diabetes mellitus patients and controls

There was no significant difference in the prevalence of *H. pylori* in cases and controls (x2 = 0.41, p = 0.52). The results therefore show that *H. pylori* infection is not significantly associated with type 2 diabetes mellitus in this study population.

Ugwu et al. [10] had reported similar findings from the South East region of Nigeria as there was no significant association between the infection prevalence in their diabetic population and non-diabetic controls(35% vr 28% respectively, p = 0.432). The findings are also in keeping with the results from various other regions of the world both developed-Australia [8], Italy [9] and developing-China [14], Turkey [15], Romania [16].

However, there exists in scientific literature, other conflicting data as reported by several investigators. In 1989, Simon et al. [4] reported that infection rates were significantly higher in diabetics than in their controls. This was the first report to examine for a possible relationship between *H. pylori* and DM. It used rapid urease test to detect infection but a major confounder, age, was not adjusted for in comparison between the groups of patients and controls.

Current thinking about the natural history of *H. pylori* infection is that childhood remains the most important period of acquisition and that spontaneous clearance of infection occurs with increasing frequency as time progresses [17,18]. Hence, the role of age in this issue is quite prominent and it should have been adjusted for before coming to conclusions about any hypothesized association. Another report that supported the positive association was from Oldenburg et al. [19]. A review of this seropositivity-based paper reveals that there was a significant difference between the ages of the diabetics and that of the controls as has also been noted by Xia et al. [8]. Thus, drawing conclusions without adjusting for this important variable may have led to the conclusion of Oldenburg et al. [19] (especially as their own analysis had showed a significantly higher prevalence was observed only in the 60–70 year olds).

The influence of age cannot be overemphasized as for instance, the initial significantly less prevalence of *H. pylori* between the type 1 diabetics and their type 2 counterparts and even those of controls disappeared when appropriate adjustments for age were made in a large Australian study [8].

Poor socioeconomic status, living conditions, and hygiene have been repeatedly demonstrated to be major risk factors for *H. pylori* infection [9,14]. Dore et al. [9] has also observed that the studies that found an association between DM and *H. pylori* infection often did not correct for this very important confounding variable. Moreover, it must be noted that the research tool employed for the detection of *H. pylori* seropositivity in the Oldenburg et al study was only 79% specific and this cannot be considered acceptable by current standards [11].

### Local *H. pylori* infection prevalence data as compared with study findings

*H. pylori* prevalence data from local studies vary widely depending on the population studied and mode of bacterial identification.

Seroprevalence studies predominate and their values vary based on the population studied and mode of *H. pylori* diagnosis. The highest and lowest prevalence values were studies in which the diagnosis of *H. pylori* was made via histology in patients undergoing endoscopy. Eighty-seven percent (78%) was the highest quoted figure and this was from a hospital-based study among Maiduguri dyspeptics [20] and the lowest figure of 22.4% was an Ibadan report from among patients with chronic gastritis [21].

The prevalence figures from this index population (18% in T2DM patients and 13% in controls) was rather low as compared to the other prevalence data as indicated above. The reasons may not farfetched.

First, it is not appropriate to compare the prevalence data from community-based and hospital-based studies as there remains the inbuilt tendency for bias in doing so. Various factors which come to play in hospital-based studies cannot be easily corrected for during data analysis. Also the hospital-base from which the study participants are drawn lends itself to the explanation that there is a higher likelihood of these patients being exposed to repeated antibiotics that might have anti-helicobacter effects [22]. The mode of diagnosis is also an issue as serology-based studies cannot compensate for the inability of the tests to distinguish between current and previous infection [23]. The stool antigen detection basis of this study represents an attempt at more accurate detection of prevalence of current *H. pylori* infection in our local population. This was also corroborated by Smith et al [24] in their study of the use of stool antigen test to diagnose *H. pylori* infection.

### *H. pylori* infection and duration of diabetes

This study does not agree with the suggestion that the prevalence of *H. pylori* infection increases with the duration of diabetes. There was no relationship between duration of DM and *H. pylori* infection. This was in agreement with findings from both local [10] and international literature [8,9,15]. There still exists controversy in this area as some researchers have noted a significant relationship between *H. pylori* infection and DM duration [5,25] while others have noted a negative relationship [22].

The Italian study [25] showed that the prevalence of *H. pylori* infection was 23%, 32%, and 40% in patients with type 1 diabetes who had a disease duration of >1 year, 1–3 year, or >3 year, respectively [26]. The age factor, again, appears to have confounded the results of these studies.

The mean ages in the above mentioned groups were 22, 30, and 37 years, respectively, and it is likely that any association between *H. pylori* infection and duration of DM would disappear if age was adjusted for in the final analyses.

The negative association report [22] was seroprevalence- based and conducted among type 1 diabetics from Spain. The seroprevalence of *H. pylori* infection was 43% in patients with the duration of <3 yr and the rate decreased to 16% for those with a longer duration. The reason adduced for this was that infection decreases during the course of DM disease because of repeated antibiotic therapy.

This study does not support the hypothesis that patients with DM are more frequently infected by *H. pylori* over time.

## Conclusions

The lack of a statistical significant difference between the *H. pylori* infection rates in T2DM patients and controls suggests that the infection is not increased in T2DM. More empowered and larger studies need to be conducted to confirm the study findings and further elucidate the relationship of these two factors- T2DM and *H. pylori* infection.

## Competing interests

The authors declare no conflict of interests in the publication of this document.

## Authors’ contributions

EA was the initiator of the research topic. He also provided for the materials for the study. AO acted as the principal investigator in this project. SS and OF gave expert input all through the research and contributed to the final editing of this publication particularly from Microbiology and Endocrinology stand points. All authors read and approved the final manuscript.
